# De Novo Vesicoureteral Reflux Following Ureterocele Decompression in Children: A Systematic Review and Meta-Analysis Comparing Laser Puncture versus Electrosurgical Incision Techniques

**DOI:** 10.3390/children9010010

**Published:** 2021-12-24

**Authors:** Sachit Anand, Tanvi Goel, Apoorv Singh, Nellai Krishnan, Prabudh Goel, Devendra Kumar Yadav, Minu Bajpai

**Affiliations:** 1 Department of Pediatric Surgery, Kokilaben Dhirubhai Ambani Hospital, Mumbai 400053, India; drsachit_anand@outlook.com; 2 Department of Pediatric Surgery, All India Institute of Medical Sciences, New Delhi 110029, India; tanvibansal510@gmail.com (T.G.); dr.singhapoorv@gmail.com (A.S.); nellai93@gmail.com (N.K.); prabudh.aiims@gmail.com (P.G.); drdevendra@hotmail.com (D.K.Y.)

**Keywords:** ureterocele, de novo vesicoureteral reflux, laser puncture, electrosurgical incision, endoscopic transurethral incision, minimally invasive surgery, children

## Abstract

Background: The available endoscopic techniques for ureterocele decompression include laser puncture (LP), electrosurgical incision (ES), and cold-knife incision. This systematic review was performed to compare the efficacy of LP versus ES techniques with special emphasis on de novo VUR. Methods: Four databases were systematically searched by the authors. The inclusion criteria were all comparative studies in which ureterocele decompression was performed by either LP or ES endoscopic techniques. Outcomes including the incidence of de novo VUR, the need for endoscopic retreatment of the ureterocele, and the need for secondary surgical procedures were studied. Risk ratios (RR) were calculated for all outcomes and the Mantel-Haenszel method was utilized for the estimation of pooled RR. The methodological quality was assessed by the Downs and Black scale. Results: Five studies were considered for systematic review, while four of them were included in the meta-analysis. Out of 202 children, 67 developed de novo VUR. Significantly lower rates of reflux were observed in the LP group vis-a-vis ES group (RR = 0.17, 95% CI 0.09 to 0.32, *p <* 0.00001). Endoscopic retreatment rates (*n* = 20) demonstrated no significant difference among the two patient groups (RR = 0.66, 95% CI 0.26–1.68, *p* = 0.38). A total of 46 secondary procedures were performed in 170 children, mostly ureteral re-implantations, with a significantly lower need of secondary surgeries following LP versus ES (RR = 0.26, 95% CI 0.13–0.49, *p <* 0.0001). The risk of bias in the included studies was low-to-moderate. Conclusions: When compared to the ES technique, the LP technique is associated with a significantly low incidence of de novo VUR and requirement for secondary surgeries (particularly anti-reflux surgeries). Endoscopic retreatment rates showed no significant difference between the two techniques. However, due to the moderate risk of bias in two out of four included studies, randomized controlled trials are needed in the future.

## 1. Introduction

A ureterocele is an abnormal cystic dilatation of the submucosal intravesical portion of the ureter [1]. The incidence of ureterocele is highly variable as per historical reports ranging from 1:5000 to 1:12,000 [2,3]. It occurs predominantly in children younger than 2 years of age and shows a female gender preponderance (M:F = 1:5) [1]. Recent advances in prenatal imaging have led to a left-shift in the diagnosis of ureterocele, with the majority of cases being diagnosed during the antenatal period [4]. Various classification systems have been proposed for ureteroceles in the past [5]. To eliminate any ambiguity, a standardized classification and nomenclature were provided by Glassberg et al. [6], which divides this anomaly into ectopic and intravesical. In addition to this, ureteroceles are commonly classified based on the type of the drainage system in the ipsilateral side, i.e., single or duplex system ureteroceles [1].

The basic principles of the management of this anomaly include optimal deroofing of the ureterocele to relieve the obstruction, minimizing the incidence of de novo vesicoureteral reflux (VUR), and prevention of subsequent urinary tract infections (UTI) [7]. In addition, due to the presence of associated pathologies in the ipsilateral (non-functioning upper moiety in a duplex system) and/or contralateral (VUR) renal units, the overall surgical morbidity needs to be minimized in these cases. Endoscopic transurethral decompression of ureteroceles has tremendously reduced surgical morbidity in these patients [7]. All the available endoscopic techniques for ureterocele decompression including laser puncture (LP), electrosurgical incision (ES), and cold-knife incision have comparable success rates in terms of the optimal deroofing or the need for redo-decompression [8]. However, the incidence of de novo VUR varies among these techniques. Ureteroceles managed via the LP technique are associated with a significantly lower incidence of de novo VUR as compared to those treated via ES [8,9,10,11]. Therefore, the use of the LP technique not only prevents UTIs by limiting vesicoureteral reflux in the ipsilateral renal unit but also provides a therapeutic advantage in terms of reducing the requirement of subsequent surgical procedures. However, the current published literature comparing the efficacy of LP versus ES techniques has a limited sample size, thereby preventing a common consensus.

This systematic review and meta-analysis were performed to systematically summarize all relevant data and define the current evidence on the comparative efficacy of LP versus ES techniques of surgical management of ureteroceles. We hypothesize that the LP technique is superior to ES in terms of the occurrence of de novo VUR and the need for secondary procedures.

## 2. Methods

### 2.1. Search Process

This systematic review was registered in the International Prospective Register of Systematic Reviews (PROSPERO). The literature search was performed as per the Preferred Reporting Items for Systematic Reviews and Meta-Analyses (PRISMA) guidelines [12]. On 7 November 2021, the PubMed database was independently explored by two authors (SA and NK) to confirm the paucity of published systematic reviews on this topic. After this preliminary search, both the authors conducted a systematic literature search in the PubMed, EMBASE, Web of Science, and Scopus databases on the same day. The search keywords used were (Diathermy OR Laser OR Electrosurgery) AND (Ureterocele). A detailed search strategy is demonstrated in Appendix A. The total records were identified and duplications were removed. Subsequently, the eligibility criteria were applied to screen the remaining studies.

### 2.2. Eligibility Criteria

The inclusion criteria were: Participants—all patients, aged <18 years, who were diagnosed with ureterocele (based on clinical examination, radiological investigations, and nuclear renal scans) and required endoscopic ureterocele decompression; Intervention—laser puncture of the ureterocele; Comparison—children in whom electrosurgical incision of the ureterocele was performed; Outcomes—the proportion of children developing de novo VUR in the punctured renal unit after ureterocele decompression was the primary outcome in this review. The proportion of children requiring endoscopic re-treatments for optimal decompression of the ureterocele, and the proportion of children requiring secondary surgeries (uretero-celectomy, ureteral reimplantation, heminephrectomy, and endoscopic injection of implants/copolymers for reflux) were the secondary outcomes.

All relevant studies where the primary outcome was reported were eligible for inclusion in this review. The type of collecting system (duplex system or single system) and the location of the ureterocele (ectopic or intravesical) were not considered specific eligibility criteria. All comparative studies with incomplete data or where the outcomes of interest were not reported were excluded. Case reports, commentaries, editorials, opinion articles, conference abstracts, and review articles were also excluded.

### 2.3. Data Extraction

Data synthesis was independently performed by two investigators (PG and TG) using the Microsoft Excel spreadsheets. Data, including the name of the first author, year of publication, study design, sample size (with group-wise distribution), gender distribution, the average age at surgery, type of collecting system (single system or duplex system), and location (ectopic or intravesical), were synthesized, along with data on the abovementioned outcomes. The senior author (MB) was consulted for resolution of any disagreements among the two authors.

### 2.4. Quality Assessment

An independent assessment of the methodological quality was performed by two authors (SA and AS) using the Downs and Black scale [13]. The validated 27-point scale has four domains of assessment with minimum and maximum scores of 0 and 32 respectively. On the basis of these scores, the risk of bias was graded as high (0–15), moderate (16–23), or low (score > 23). The kappa statistics were used to identify the level of inter-rater agreement regarding the risk of bias [14]. The degree of agreement was graded as slight (0.00–0.20), fair (0.21–0.40), moderate (0.41–0.60), substantial (0.61–0.80), and almost perfect (0.81–1.00).

### 2.5. Statistical Analysis

The baseline data were expressed as numbers, proportions, averages, and ranges. RevMan 5.4 (Cochrane Collaboration, London, UK) software was used to perform the meta-analysis. The guidelines from the Cochrane handbook were followed during the course of this study [15]. As all three outcomes were dichotomous, the risk ratios (RR) were calculated for each of them. Subsequently, the Mantel-Haenszel method was utilized to calculate the pooled RR. The heterogeneity among the included studies was estimated using the I^2^ statistics. In the case of substantial heterogeneity (I^2^ > 50%), a random-effects model was used. A *p*-value of <0.05 was considered statistically significant. Children were divided into two treatment groups, i.e., LP and ES, consisting of those managed via the laser puncture and electrosurgical approaches, respectively.

## 3. Results

### 3.1. Characteristics of the Included Studies

**Figure 1:**
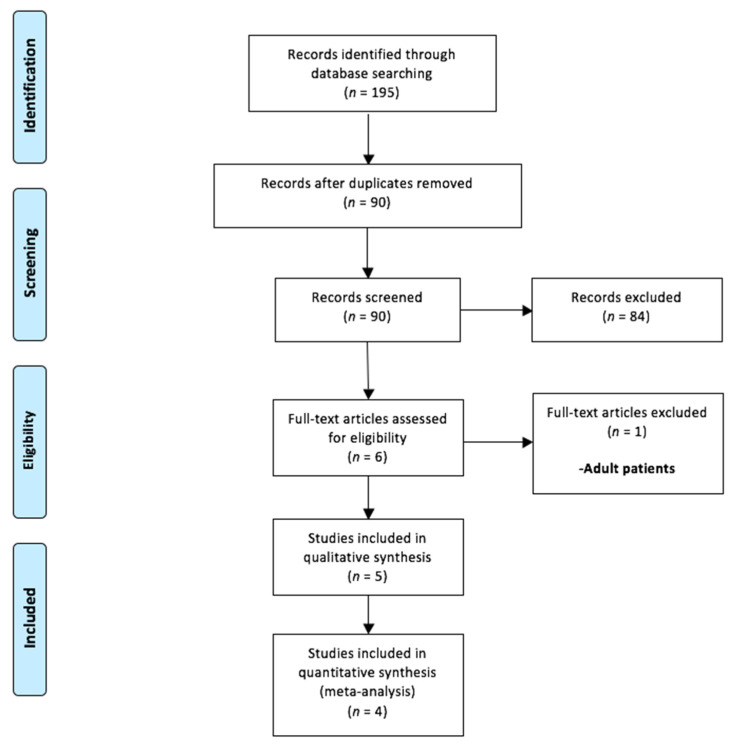
Selection of relevant studies using the Preferred Reporting Items for Systematic Review and Meta-Analysis (PRISMA) flow diagram.

A total of 195 records were identified with our search strategy (Figure 1). Out of these, 105 duplications were removed. The remaining 90 abstracts were screened as per the inclusion/exclusion criteria. Of these, 84 abstracts were excluded. Only six full-texts were considered relevant for eligibility [7,8,9,10,11,16]. One of these was further excluded as it consisted of adult patients [16]. Therefore, five studies were considered for systematic review [7,8,9,10,11], while four were included in the final meta-analysis [8,9,10,11]. The study by Haddad et al. [7] was considered for the meta-analysis because it compared the watering-can method of LP of ureterocele versus all the conventional methods (including incision using cold-knife and ES incision).

The baseline characteristics of the included studies are demonstrated in Table 1. All four included studies had a retrospective study design. A total of 202 patients, 106 and 96 belonging to the LP and ES groups, respectively, were included in the analysis. There was a clear female preponderance among these patients. Although the average age at surgery was non-uniform among the included studies, none of these studies demonstrated a significant difference in age at surgery among the two treatment groups. The type of collecting system was depicted by three studies [9,10,11], and the majority of the included subjects had a duplex system (in the renal unit subtending the ureterocele). The location of the ureterocele, whether intravesical or ectopic, was shown by all four studies [8,9,10,11]. While two of them highlighted a majority of ectopic ureteroceles [9,10], the remaining two studies included only intravesical ureteroceles [8,11].

### 3.2. Summary of the Included Studies

#### 3.2.1. Pogorelić et al., 2021

This study from Croatia included a total of 64 neonates with intravesical ureterocele, 41 and 23 belonging to the LP and ES groups, respectively. Within the LP group, the ureterocele puncture was performed utilizing a holmium laser fiber (20 W Holmium laser) at 6 Hz and 0.6 J. Six to eight punctures were made in the ureterocele creating a watering-can appearance. Optimal ureterocele decompression was achieved in 100% of the children from the LP group versus 88% of those belonging to the ES group. Endoscopic re-treatment was required in five patients from the ES group, while none of the children from the LP group required re-deroofing. Postoperatively, ultrasound (USG) was performed at the first postoperative day, one month, six months and then once every year. Voiding cystourethrogram (VCUG) or contrast-enhanced uro-sonography (ce-VUS) was also performed at six months follow-up or after febrile UTI. As compared to the ES group, the incidence of de novo VUR was significantly lower in the LP group. The need for secondary procedures was also significantly more among the children belonging to the ES group [8].

#### 3.2.2. Di Renzo et al., 2020

This retrospective study from Italy compared the outcomes of children treated via the LP and ES approaches of ureterocele decompression. Within the LP group, the holmium:yttrium-aluminum-garnet (Ho:YAG) laser was used at a setting of 6–8 Hz and 0.6–0.8 J to make multiple punctures (4–8) on the ureterocele. Laser fibers of 550 micron were used in all patients except the first where a 200 micron fiber was used. A total of seven and nine children were recruited in LP and ES groups, respectively. The follow-up protocol included USG and VCUG (or VUS) at 3 months follow-up for all patients, and subsequently in cases of persistent de novo VUR. The occurrence of de novo VUR and the need for endoscopic re-treatment did not differ among the two treatment groups. However, when compared to the ES group, VUR spontaneously resolved among the patients belonging to the LP group. The need for secondary surgeries was also significantly lower among the children belonging to the LP group [9].

#### 3.2.3. Caione et al., 2019

This study was conducted in Italy and included 90 children. Of these, 64 and 26 belonged to the LP and ES groups respectively. The Ho:YAG laser was used at a setting of 0.5–0.8 J with a frequency of 5–9 Hz during ureterocele decompression for the patients belonging to the LP group. Laser fibers of 272 and 550 micron were used based on the surgeon’s preference. Four to ten laser punctures were made at the ureterocele base. Successful ureterocele decompression was achieved in 92% of the patients from each group. During follow-up, USG was performed at 1, 3, 6 months and then once every year. VCUG was reserved for cases with culture-proven UTI and those showing significant upper tract dilatation without decompressive changes at 6–12 months. No significant difference was observed among the two treatment groups in terms of the need for endoscopic re-treatment. The occurrences of febrile UTI and de novo VUR were significantly lower among the children belonging to the LP group versus the ES group. In addition, the need for secondary surgeries was significantly less among the former [10].

#### 3.2.4. Ilic et al., 2018

This retrospective study was conducted in Serbia. Out of 32 included children, 20 were treated via the LP approach (Ho:YAG laser) of ureterocele decompression. Micro laser fibers (200 and 550 micron) providing 0.2–1 J of energy at 5 Hz frequency were used to make 4–10 punctures on the ureterocele wall. The follow-up protocol included USG evaluation at the first postoperative day, one month, and three months follow-up. VCUG was mandatory for all patients within the ES group. For the LP group, VCUG was performed only in cases of UTI and upper tract dilatation on USG. The occurrence of de novo VUR was significantly lower among the children belonging to the LP group versus the ES group (8.3% versus 65%, *p* = 0.003). The endoscopic retreatment rate showed no significant difference among the two treatment groups [11].

### 3.3. Quality Assessment

The Downs and Black scores assigned to each study are shown in Table 2. The average scores ranged from 19 to 26. The studies by Di Renzo et al. [9] and Pogorelić et al. [8] had the minimum and maximum scores, respectively. The risk of bias in these studies was graded as low-to-moderate. The inter-observer agreement for quality assessment was almost perfect (kappa = 0.954; *p* < 0.001).

### 3.4. Outcome Analysis

#### 3.4.1. De Novo Vesicoureteral Reflux

**Figure 2:**
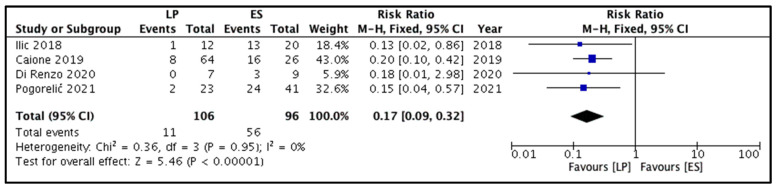
Forest plot comparison between the two patient groups in terms of the incidence of de novo vesicoureteral reflux. Legends: LP, laser puncture patient group. ES, electrosurgical incision patient group. M-H, Mantel-Haenszel method. CI, confidence interval.

The proportion of children developing de novo reflux in the punctured renal unit following ureterocele decompression was reported by all four studies [8,9,10,11]. Out of 202 children, 67 developed de novo reflux. Of these 67, 11 and 56 belonged to the LP and ES groups, respectively. Pooling the data (Figure 2) demonstrated significantly lower rates of de novo reflux in children belonging to the LP group versus the ES group (RR = 0.17, 95% CI 0.09 to 0.32, *p <* 0.00001). For this outcome, the heterogeneity could not reach statistical significance (I^2^ = 0%, *p* = 0.95).

The grades of de novo VUR were depicted in three studies only [8,9,11]. Out of 43 children with de novo VUR in these studies, 33 had high-grade reflux (grade IV–V). All these 33 children belonged to the ES treatment group. On the other hand, none of the children from the LP group developed high-grade de novo reflux.

#### 3.4.2. Endoscopic Retreatment for Ureterocele Decompression

**Figure 3:**
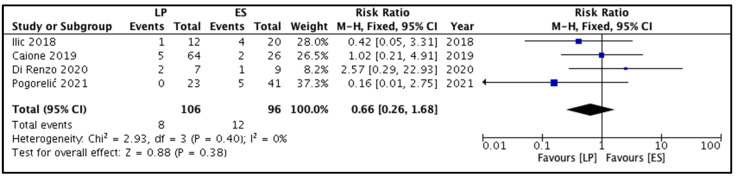
Forest plot comparison between the two patient groups in terms of the requirement of endoscopic retreatment for ureterocele decompression. Legends: LP, laser puncture patient group. ES, electrosurgical incision patient group. M-H, Mantel-Haenszel method. CI, confidence interval.

All four studies reported this outcome [8,9,10,11]. Repeat endoscopic decompression was performed in 20 out of 202 children. Of these 20 children, 8 and 12 belonged to the LP and ES groups, respectively. The pooled risk ratio (Figure 3) for the need for endoscopic re-treatment among children belonging to the LP group versus the ES group was 0.66 (95% CI 0.26 to 1.68), showing no significant difference (*p* = 0.38). The estimated heterogeneity among the included studies could not reach statistical significance (I^2^ = 0%, *p* = 0.40) for this outcome.

#### 3.4.3. Secondary Procedures

**Figure 4:**
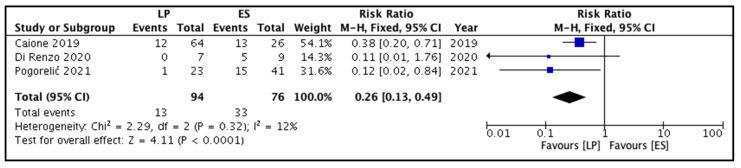
Forest plot comparison between the two patient groups in terms of the requirement for secondary surgical procedures. Legends: LP, laser puncture patient group. ES, electrosurgical incision patient group. M-H, Mantel-Haenszel method. CI, confidence interval.

Three studies reported this outcome. A total of 46 secondary procedures were performed in 170 children [8,9,10]. Secondary procedures performed for the LP and ES treatment groups were 13 and 33, respectively. The majority of these were ureteral re-implantations. Endoscopic injections of implant/copolymer were depicted by one study only [8]. Pooling the data (Figure 4) demonstrated a significantly lower need for secondary procedures following LP versus ES (RR = 0.26, 95% CI 0.13 to 0.49, *p <* 0.0001). For this outcome, the estimated heterogeneity among the included studies could not reach statistical significance (I^2^ = 12%, *p* = 0.32).

**Figure 5:**
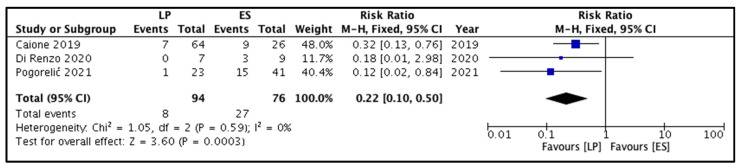
Forest plot comparison between the two patient groups in terms of the requirement for anti-reflux surgeries (excluding other secondary procedures). Legends: LP, laser puncture patient group. ES, electrosurgical incision patient group. M-H, Mantel-Haenszel method. CI, confidence interval.

For this outcome, a separate exclusion analysis was also performed (Figure 5) where non-anti-reflux surgeries were excluded. This pooled analysis also showed that the number of subsequent surgical procedures was significantly less among the children belonging to the LP treatment group versus the ES group.

## 4. Discussion

The management of ureterocele depends upon various factors including the age of the child, his/her clinical condition, the location of the ureterocele, the type of drainage system on the side subtending the ureterocele, split function of both the kidneys and both the moieties (in a duplex system), the grade of preoperative VUR (if any) in the lower moiety, etc. [4]. In addition, follow-up is not always guaranteed in resource-challenged nations and high rates of treatment default are likely. Considering this, it is evident that multiple reasons are responsible for the lack of a standardized approach for ureterocele management. While some surgeons follow a proactive approach and perform upfront uretero-celectomy with lower tract reconstruction, others propose a wait-and-watch approach following endoscopic deroofing of the ureterocele [4].

The results of the present meta-analysis highlighted the superiority of the LP technique over the ES technique for ureterocele decompression. The incidence of de novo VUR was significantly low among children undergoing LP versus ES of the ureterocele. Similar results have been depicted by Haddad et al. [7], where the incidence of de novo VUR in the LP group was less than half as compared to the ES group (32% versus 67%; *p* < 0.05). In a preliminary experience by Palmer et al. [17], a similar therapeutic advantage of LP of ureterocele was highlighted. In their study, the incidence of de novo reflux in LP versus ES techniques was 36% and 88%, respectively, demonstrating a statistically significant difference.

A higher incidence of de novo VUR in the ES group is mainly because of the creation of large-caliber defects in the ureteroceles [5,7]. These large-sized defects tend to impair the flap-valve anti-reflux mechanism, which is pivotal in preventing the VUR during bladder filling [18]. In contrast, the smaller puncture holes created during LP of the ureterocele do not interfere with this anti-reflux mechanism. It must also be mentioned that grades of de novo VUR were different among the two patient groups in the present analysis [8,9,11]. While children belonging to the ES group had persistent high-grade de novo VUR, the subjects in the LP group had low-grade reflux that would resolve spontaneously.

Another advantage of the LP technique is less requirement for subsequent surgical procedures. In this meta-analysis, a significant difference was observed among the two treatment groups in terms of the need for secondary surgical procedures. It is noteworthy that the definition of secondary surgeries also includes procedures performed for associated anomalies (e.g., non-functioning upper moiety in a duplex system, etc.). Therefore, to eliminate this bias, exclusion analysis (excluded all procedures other than anti-reflux surgeries) was performed. This separate meta-analysis also showed consistent findings of a significantly less requirement for subsequent anti-reflux procedures in the LP group versus the ES group. It is believed that these findings are secondary to the significant difference of de novo reflux among the two treatment groups.

In this review, the success rates of both techniques were also compared by a pooled analysis of endoscopic retreatment rates. No significant difference among the two groups demonstrated comparable efficacy in terms of optimal relief of obstruction. Similar findings have been reported in the available literature. Haddad et al. showed ureterocele decompression rates of almost 90% with either approach [7]. In the same study, irrespective of the type of technique, a consistent improvement in hydronephrosis was depicted during the postoperative period. Pogorelić et al. [8] have also reiterated that all the endoscopic techniques are equally effective in relieving the obstruction.

The minimum number of holes required for adequate decompression of the ureterocele during the LP technique is not known and is still a matter of debate. While Pogorelić et al. [8] propose 6–8 small-sized laser-created holes, Ilic et al. [11] have shown that 4–10 punctures in the ureterocele wall are sufficient. Haddad et al. [7] have also highlighted the benefits of multiple small holes in their study. Their technique, named as the watering-can technique, describes the creation of 10–20 small caliber holes using laser fiber. The advantage of this technique is unabated urine flow across the vesico-ureteric junction during the postoperative period as some of these holes invariably undergo re-epithelialization.

There are a few limitations of this study. First, the sample size of the included studies is limited. Second, the retrospective nature is a source of information bias due to variable reporting. Three out of four studies depicted the requirement of secondary surgeries. The baseline characteristics were also non-uniformly reported. Three and two studies described the type of collecting system and location of the ureterocele respectively. In addition, the grades of de novo VUR were reported by three studies only [8,9,11]. Thirdly, this meta-analysis involves the pooling of the data from a heterogeneous group of patients in terms of the age at ureterocele decompression, the location of ureterocele, and the type of collecting system. It has been demonstrated that both ectopic ureteroceles and the ureteroceles associated with the duplex system are more likely to require secondary surgeries [4]. Finally, these patients were managed by different surgeons practicing in different countries. Outcome differences can arise because of differences in the surgeon’s experience and the lack of standardized approach among them. In addition, a variable follow-up period among the included studies can also be a source of bias.

Despite these limitations, the present meta-analysis is the first to study the outcomes of LP and ES techniques of ureterocele decompression in terms of the incidence of de novo VUR, the requirement of endoscopic re-deroofing, and the requirement of secondary surgeries. As per the data provided by the current published studies, endoscopic decompression performed by the LP approach is superior to the ES approach. The strengths of the present review include reporting and external validity while the weaknesses lie in internal validity and power.

## 5. Conclusions

When compared to the ES technique, endoscopic decompression of the ureterocele performed via the LP technique is associated with a significantly low incidence of de novo VUR. The requirement of secondary surgeries (and anti-reflux surgeries) was also significantly less among the LP group. The need for endoscopic retreatment of ureteroceles demonstrated no significant difference among the two treatment groups. However, two out of four included studies had a moderate risk of bias. Therefore, further randomized controlled trials need to be conducted before any definite conclusions are drawn.

## Figures and Tables

**Table 1:** Characteristics of included studies.

First Author, Year of Publication	Sample Size		No of Females (%)		Average Age at Surgery; in Days		Type of Collecting System (% Duplex)		Location (% Ectopic)	
	LP	ES	LP	ES	LP	ES	LP	ES	LP	ES
Pogorelić et al., 2021	23	41	16 (70)	28 (68)	12.5 (8–28) ^§^	11 (7–26) ^§^	Not mentioned		All intravesical	
Di Renzo et al., 2020	7	9	7 *	6 (67)	92 (17–160) ^§^	37 (7–111) ^§^	71	100	71	67
Caione et al., 2019	64	26	55 (86)	21 (81)	6.3 mo (1–168) ^†^	5.9 mo (1–123) ^†^	83	85	77	81
Ilic et al., 2018	12	20	8 (67)	14 (70)	9.8 (4–28) ^§^	10.2 (6–28) ^§^	75	80	All intravesical	

* All were female; ^§^ Mean (range); ^†^ Median (range); Abbreviations: LP, laser puncture group. ES, electrosurgical incision group. mo, in months.

**Table 2:** Downs and Black scale scores assigned to each included study by both observers.

Scoring by Observer 1						
Study	Reporting	External Validity	Internal Validity-Bias	Internal Validity-Confounding	Power	Total Scores
Pogorelić et al., 2021	10	3	5	3	5	26
Di Renzo et al., 2020	8	3	5	3	0	19
Caione et al., 2019	8	3	5	3	5	24
Ilic et al., 2018	9	3	5	3	2	22
**Scoring by Observer 2**						
**Study**	**Reporting**	**External Validity**	**Internal Validity-Bias**	**Internal Validity-Confounding**	**Power**	**Total Scores**
Pogorelić et al., 2021	10	3	5	3	5	26
Di Renzo et al., 2020	8	3	5	3	0	19
Caione et al., 2019	7	3	5	3	5	23
Ilic et al., 2018	10	3	5	3	2	23
**Inter-Observer Agreement**						
**Study**	**Observer 1**	**Observer 2**	**Mean**	**Kappa Value**	***p***	
Pogorelić et al., 2021	26	26	26	0.954	<0.001	
Di Renzo et al., 2020	19	19	19			
Caione et al., 2019	24	23	23.5			
Ilic et al., 2018	22	23	22.5			

## Data Availability

The data presented in this study are available upon request of the respective author.

## Appendix A

**PubMed**—(((Diathermy) OR (Laser)) OR (Electrosurgery)) AND (Ureterocele)
**EMBASE**—(‘diathermy’:ti,ab,kw OR ‘electrosurgery’:ti,ab,kw OR ‘laser’:ti,ab,kw) AND ureterocele:ti,ab,kw)
**Web of Science**—Query 1: ((ALL = (diathermy)) OR ALL = (electrosurgery)) OR ALL = (laser) **AND** Query 2: ALL = (ureterocele)
**Scopus**—(TITLE-ABS-KEY(diathermy OR electrosurgery OR laser) AND TITLE-ABS-KEY (ureterocele)

**Database**	**Studies**
PubMed	55
EMBASE	26
Web of Science	36
Scopus	78
Total	195
Duplications	105
After duplication removal	90
